# Diagnostic Value of Preoperative Electrodiagnostic Analysis in a Patient with Facial Palsy and a Large Vestibular Schwannoma: Case Report

**DOI:** 10.3390/diagnostics12020542

**Published:** 2022-02-20

**Authors:** Myung Chul Yoo

**Affiliations:** Department of Physical Medicine and Rehabilitation, College of Medicine, Kyung Hee University Hospital, 23 Kyung Hee Dae-ro, Dongdaemun-gu, Seoul 02447, Korea; famousir@naver.com; Tel.: +82-2-958-8980; Fax: +82-2-958-8470

**Keywords:** vestibular schwannoma, electrodiagnostic study, blink reflex, needle electromyography

## Abstract

Although radiologic methods confirm the diagnosis of patients with large vestibular schwannomas, these methods usually indicate only the size of the tumor and its possible nerve compression. Electrodiagnostic methods can reveal the functional state of the nerves, particularly the trigeminal and facial nerves, as well as providing a basis for objectively evaluating nerve injury. Due to the lack of an established objective evaluation method, electrodiagnostic methods were utilized to assess injury to the cranial nerve in a patient with a large vestibular schwannoma. A 79-year-old woman presented with a one-month history of right facial palsy, vertigo, dizziness, right postauricular pain, and right-sided hearing disturbance. Physical examination suggested injuries to the facial and vestibulocochlear nerves. Magnetic resonance imaging identified a vestibular schwannoma and showed that the tumor mass was affecting the brainstem, including the fourth ventricle, resulting in mild obstructive hydrocephalus. Preoperative electrodiagnostic evaluation identified asymptomatic trigeminal neuropathy accompanying a vestibular schwannoma. The patient underwent surgery, consisting of a suboccipital craniotomy with additional gamma knife radiosurgery. Postoperatively, she demonstrated significant recovery from right facial palsy and partial improvement of her neurologic symptoms. Large vestibular schwannomas with facial paralysis may be accompanied by additional entrapment neuropathy. Routine preoperative electrophysiological evaluation is recommended to establish a definitive diagnosis and evaluate the function of the trigeminal nerve, facial nerve, and brainstem in patients with large and compressive vestibular schwannomas.

## 1. Introduction

Vestibular schwannomas, also called acoustic neuromas, are benign, slow-growing tumors that typically arise from the Schwann cells that form the vestibular portion of the vestibulocochlear nerve sheath [1]. These tumors are relatively common, accounting for 6–8% of all intracranial tumors and 80% of cerebellopontine angle (CPA) tumors [2,3]. Symptoms are usually associated with the compression of adjacent cranial nerves, the brainstem, and/or posterior fossa structures [4]. Although the most common symptoms of vestibular schwannoma are typically of insidious onset, other symptoms, including hearing deficits, tinnitus, vertigo, and dizziness, can occur. In addition, large tumors may compress the trigeminal nerve, resulting in trigeminal disturbances, such as hyperesthesia, paresthesia, and neuralgia. As a vestibular schwannoma grows, it can expand from its origin within the internal auditory canal (IAC) and extend into the brainstem or the CPA. The tumor can continue to enlarge and compress nearby cranial nerves. Clinically apparent facial paralysis is rare, occurring in fewer than 5% of patients with vestibular schwannoma, usually late in the course of disease [5,6]. In addition, a study of 1000 patients with vestibular schwannomas showed that 17% experienced trigeminal nerve disturbances, with major symptoms that included facial numbness (paresthesia), hypoesthesia, and pain [7].

Vestibular schwannomas must be large to affect the trigeminal or facial nerve. As the diagnosis is based on clinical findings, trigeminal nerve damage is not suspected unless patients report major symptoms, such as trigeminal neuralgia. At present, medical imaging, particularly magnetic resonance imaging (MRI), and physical examination are primarily used in the differential diagnosis of patients with neurological symptoms. Most vestibular schwannomas can be preoperatively diagnosed by MRIs that demonstrate tumor extension beyond the fundus into the proximal fallopian canal. Imaging has become crucial in initial screening and evaluation and can precisely characterize vestibular schwannomas, enabling surgical planning for their removal. However, the correlations of symptoms and tumor extensions with actual objective cranial nerve damage remain unclear, as are the ability to preoperatively determine the extent of damage to the cranial nerves and the clinical significance of this damage. As no other dependable diagnostic tools have emerged to evaluate nerve injuries, surgical exploration remains the only definitive method for establishing a diagnosis. Electrodiagnostic techniques, including evaluations of nerve conduction and blink reflex, and needle electromyography (EMG), may be objective and noninvasive methods for evaluating nerve injury in patients with vestibular schwannomas. This study describes the use of electrodiagnostic techniques to predict damage to the trigeminal and facial nerves in a patient with a large vestibular schwannoma.

## 2. Case Presentation

**Figure 1:**
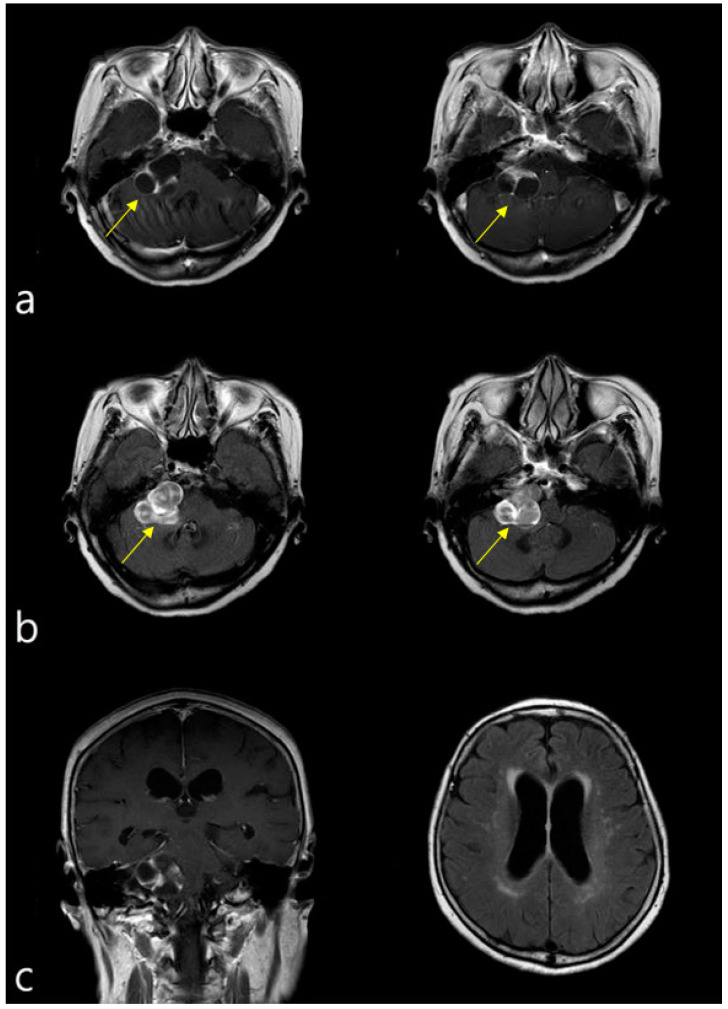
Brainstem magnetic resonance imaging (MRI). (**a**) T1-weighted enhanced imaging showing a well-margined, enhancing mass at the right cerebellopontine angle (3.4 × 2.7 cm), compatible with vestibular schwannoma (arrows). (**b**) T2-weighted MRI imaging, showing high signal intensity similar to CSF, demonstrating heterogeneous enhancement (arrows). (**c**) Image showing compression by the tumor mass of the right side of the brainstem, resulting in mild obstructive hydrocephalus.

A 79-year-old woman visited the outpatient clinic of the Department of Otorhinolaryngology, Head and Neck Surgery of our university hospital with a 1-month history of right facial palsy, vertigo, dizziness, right postauricular pain, and right-sided hearing disturbance. Her medical history included bipolar hemiarthroplasty due to a right hip fracture, including total arthroplasty of both knees, in 2017. She had no history of neurological, psychological, or metabolic disorders. Physical and neurologic examinations revealed marked right peripheral facial palsy (House-Brackmann grade IV), gaze-evoked nystagmus, and cerebellar ataxic gait. No significant facial sensory deficit was observed. Based on her symptoms, injuries to her facial and vestibulocochlear nerves were suspected. Brain MRI showed a solid cystic mass with heterogeneous enhancement in the right CPA and IAC. The tumor measured 3.4 × 2.7 cm in size, with thin walls and high signal intensity similar to cerebrospinal fluid (CSF) on T2-weighted MRI images and low signal intensity on T1-weighted MRI images (Figure 1a,b). The extension of the tumor and brainstem distortion resulted in narrowing of the fourth ventricle and mild obstructive hydrocephalus due to the effect of the mass on the brainstem (Figure 1c).

The patient was referred to the Department of Physical Medicine and Rehabilitation for electrodiagnostic analysis to determine the extent of facial nerve injury and to plan surgical treatment. Electrodiagnostic methods included a nerve conduction study (NCS), assessment of blink reflex, and needle EMG, all of which were performed by a physical medicine and rehabilitation physician. The facial NCS recorded the latency and amplitude of the compound muscle action potential (CMAP) of the bilateral frontalis, orbicularis oculi, nasalis, and orbicularis oris muscles in response to stimulation of the temporal, zygomatic, and buccal branches of the facial nerve, respectively. The facial NCS showed delayed latency in all right-sided muscles, except for the orbicularis oculi, as well as a small CMAP amplitude in all right-sided muscles (Table 1).

**Figure 2:**
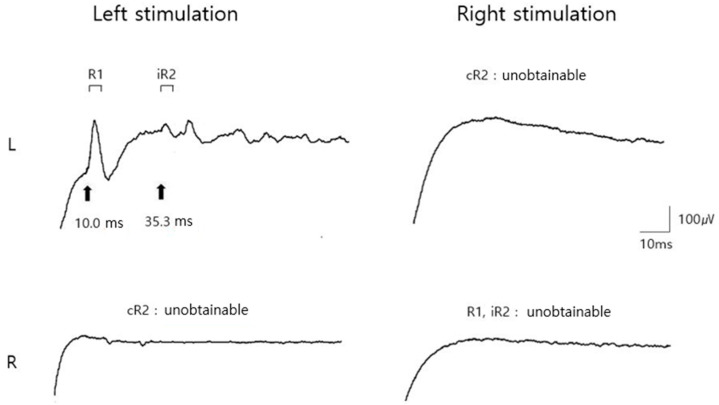
Preoperative blink reflex study results. Stimulation of the left supraorbital nerve provoked normal ipsilateral early wave (R1) and late wave (R2) responses, however did not provoke a contralateral R2 response. Stimulation of the right supraorbital nerve did not provoke ipsilateral R1 and R2 and contralateral R2 responses. Abbreviations: iR2, ipsilateral R2; cR2, contralateral R2.

The blink reflexes of the ipsilateral R1 and R2, and contralateral R2 potentials in response to stimulation of the supraorbital nerves bilaterally were recorded by surface electrodes placed over the orbicularis oculi muscles. Although left supraorbital nerve stimulation provoked normal ipsilateral R1 and R2 responses, it did not provoke a contralateral R2 response. In contrast, right supraorbital nerve stimulation did not induce ipsilateral R1 and R2 or contralateral R2 responses, indicating deficits in the afferent pathway of the right trigeminal nerve and the efferent pathway of the reflex arc, indicative of the facial nerve (Figure 2). A needle EMG examination of the bilateral frontalis, orbicularis oculi, nasalis, and orbicularis oris muscles innervated by the facial nerve was also performed. As right supraorbital nerve stimulation failed to produce potentials on either side, an additional needle EMG study of the bilateral masseter and temporalis muscles was performed to evaluate concomitant trigeminal neuropathy. All the right-sided muscles except for the nasalis muscle demonstrated fibrillation potentials, positive sharp waves, and abnormal motor unit action potential (MUAP) recruitment patterns, indicating subacute axonal loss (Table 1). All the left-sided muscles were normal on both the NCS and needle EMG examinations. Therefore, electrodiagnostic methods were able to confirm injury to both the trigeminal and facial nerves, allowing accurate documentation of the degree of axonal loss clinically in a patient with entrapment neuropathy resulting from compression by a large vestibular schwannoma.

One week later, the patient underwent retromastoid suboccipital craniotomy for tumor excision. Exposure of the lesion revealed that the tumor mass grossly compressed both the trigeminal and facial nerves. The patient experienced no postoperative complications, and postoperative histopathologic examination confirmed a vestibular schwannoma. The patient’s neurologic symptoms were partially improved after surgery. However, a brain MRI performed one month after surgery revealed a remnant tumor mass near the right IAC. The patient underwent additional gamma knife radiosurgery for treatment of the remnant tumor. One month after gamma knife radiosurgery, the patient showed recovery from the right facial palsy (House–Brackmann grade II).

## 3. Discussion

Cystic vestibular schwannomas can appear on computed tomography or MRI as peripherally thin-walled tumors or centrally located thick-walled tumors. Although these tumors are benign, patients frequently experience rapid progression of facial nerve symptoms [8]. Clinical presentation is dependent on tumor size at diagnosis, the effect of the tumor mass on the brainstem, and possible obstruction of CSF pathways. In the present patient, severe brainstem displacement and fourth ventricle tumor compression resulted in mild hydrocephalus. Patients with large vestibular schwannomas, including those with stage IV tumors, therefore frequently present not only with auditory neuropathy and vestibulopathy, but also with entrapment nerve symptoms caused by pressure on the facial nerve. The incidence of symptoms and the anatomic relationship between the tumor and the respective cranial nerve have been described previously [9].

Generally, vestibular schwannoma-related trigeminal neuropathy is thought to be caused by direct pressure of the tumor on the trigeminal nerve, leading to demyelination of the somatosensory and pain fibers [5,10]. It has been estimated that 1–9.9% of patients with trigeminal neuralgia symptoms are eventually diagnosed with CPA tumors [11]. Although both cranial nerves in the present patient were directly compressed by the tumor, she lacked the classic provocative features associated with trigeminal neuropathy. Due to a lack of objective methods available to evaluate the cranial nerves, patients are usually diagnosed based on their subjective symptoms, such as facial paralysis, vertigo, dizziness, and hearing deficits. As the symptoms in the present patient were not as intense as expected for traditional trigeminal neuralgia, such as facial numbness, hypoesthesia, and pain, she was suspected of having facial and vestibulocochlear neuropathies.

Although MRI often confirms the diagnosis of cystic vestibular schwannomas, radiological findings may only indicate tumor size and the possibility of nerve compression, especially in patients with large tumors. In contrast, electrodiagnostic techniques including NCS, blink reflex, and needle EMG, can reveal the functional state of the nerves, particularly the trigeminal and facial nerves, and offer important information for the objective evaluation of nerve damage. Electrodiagnostic methods, such as NCS and EMG [12,13], have been used to assess the degree of facial nerve injury. These electrophysiologic techniques can indirectly quantify facial nerve function by recording the compound muscle action potentials (CMAPs) and motor unit action potentials (MUAPs) [14]. The degree of nerve degeneration can be estimated by comparing the peak-to-peak amplitudes of the CMAPs on the affected side with the response amplitudes on the non-affected side. Thus, extending electrophysiological assessment by evaluating the blink reflex or needle EMG of the facial muscles could indicate the actual degree of nerve and brainstem damage at the site of tumor compression [15].

Spontaneous waveforms recorded in resting muscles during needle EMG can be important in determining the type of underlying neuromuscular disorder and its severity, prognosis, and time course. Rapid insertion of the electrode into the muscle usually results in temporary electrical activity, which lasts slightly longer than the duration of electrode insertion, due to both direct physical stimulation and denervation of muscle fibers. Insertion activity usually does not last longer than 300 ms; activity that lasts more than 300 ms after the needle movement stops is abnormal and can be observed in cases of denervation, myopathy, and inflammation [16]. Damage to a nerve that innervates a muscle, as in denervation, can cause the muscle fiber to become hypersensitive and generate abnormal spontaneous discharge potentials, such as fibrillation or positive sharp waves. In general, abnormal spontaneous activity, including positive sharp waves and fibrillation potentials, suggests active denervation. This abnormality has been encountered in patients with various neuromuscular disorders that cause denervation or damage to muscle fibers [17]. In neuropathic diseases, recruitment is reduced and may be the earliest physiological sign of nerve injury. The MRI findings in the present patient showed the effect of the CPA tumor mass on the brainstem, including the fourth ventricle, resulting in mild obstructive hydrocephalus. In addition, damage to the loop of a reflex arc was evaluated by measuring the blink reflex, which can identify defects in afferent and efferent pathways. Moreover, the decreased CMAP amplitude of the facial NCS, abnormal spontaneous activity (insertion activity, fibrillation, and positive sharp waves), MUAP polyphasic pattern, and abnormal recruitment pattern of the needle EMG confirmed the subacute axonal loss of the two cranial nerves. Therefore, electrodiagnostic techniques confirmed injury to both the trigeminal and facial nerves in the present patient, indicating that electrodiagnostic methods allow accurate clinical documentation of the degree of axonal loss in patients with entrapment neuropathy resulting from compression by a large vestibular schwannoma.

Electrodiagnostic methods are generally more accurate than neurological tests in the diagnosis of facial neuropathies. For example, electrodiagnostic techniques were about five times more accurate at diagnosing facial neuropathy than clinical evaluations by neurologists [15]. Electrodiagnostic methods also allow accurate documentation of the state of the nerves and the degrees of axonal and demyelinating damage, making these methods both prognostic and diagnostic [18,19]. Interestingly, in the present patient, trigeminal neuropathy was preoperatively identified in a CPA tumor without neurological tests and symptoms of nerve compression. Electrodiagnostic methods, including evaluations of nerve conduction and blink reflex, and needle EMG, can objectively and noninvasively evaluate nerve injury in patients with vestibular schwannomas.

## 4. Conclusions

The findings in this patient indicate that additional entrapment neuropathy may be encountered in patients with large vestibular schwannomas and facial paralysis.

A routine preoperative electrophysiological evaluation can be helpful in the definitive diagnosis of nerve damage and can evaluate the function of the trigeminal nerve, facial nerve, and brainstem in patients with large and compressive vestibular schwannomas.

## Figures and Tables

**Table 1:** Results of the facial nerve conduction study and the needle EMG examination of the present patient.

Nerve and Sites	Latency (ms)			Amplitude (µm)	Duration (ms)	Area (mVms)
Rt. facial—Frontalis	3.90			246.7	3.90	1.0
Lt. facial—Frontalis	3.45			580.0	3.45	2.3
Rt. facial—Orbicularis oculi	2.85			305.0	2.85	1.2
Lt. facial—Orbicularis oculi	2.85			995.0	2.85	3.7
Rt. facial—Nasalis	3.55			188.3	3.55	0.6
Lt. facial—Nasalis	3.05			860.0	3.05	2.5
Rt. facial—Orbicularis oris	3.10			401.7	3.10	3.4
Lt. facial—Orbicularis oris	2.95			945.0	2.95	3.6
**Muscle**	**Spontaneous Activity**			**MUAP**		**Recruitment** **Pattern**
	**IA**	**Fib**	**PSW**	**Amplitude**	**Duration**	
Rt. Frontalis	normal	1+	2+	1−	normal	discrete
Lt. Frontalis	normal	none	none	normal	normal	normal
Rt. Orbicularis ocui	normal	1+	2+	normal	normal	discrete
Lt. Orbicularis ocui	normal	none	none	normal	normal	normal
Rt. Nasalis	normal	none	none	2−	normal	single unit
Lt. Nasalis	normal	none	none	normal	normal	normal
Rt. Orbicularis oris	normal	3+	3+	1−	normal	discrete
Lt. Orbicularis oris	normal	none	none	normal	normal	normal
Rt. Masseter	increased	2+	2+	2−	normal	discrete
Lt. Masseter	normal	none	none	normal	normal	normal
Rt. Temporalis	increased	2+	2+	1−	normal	reduced
Lt. Temporalis	normal	none	none	normal	normal	normal

Abbreviations: IA, insertion activity; Fib, fibrillation potentials; PSW, positive sharp waves; MUAP, motor unit action potential.

## Data Availability

Not applicable.
